# *In silico* proposition to predict cluster of B- and T-cell epitopes for the usefulness of vaccine design from invasive, virulent and membrane associated proteins of *C. jejuni*

**DOI:** 10.1186/s40203-016-0020-y

**Published:** 2016-07-04

**Authors:** Tahirah Yasmin, Salma Akter, Mouly Debnath, Akio Ebihara, Tsutomu Nakagawa, A. H. M. Nurun Nabi

**Affiliations:** Department of Biochemistry and Molecular Biology, University of Dhaka, Dhaka-, 1000 Bangladesh; Laboratory of Applied Biochemistry, Faculty of Applied Biological Sciences, Gifu University, 1-1 Yanagido, Gifu, 501-1193 Japan

**Keywords:** Epitopes, *Campylobacter jejuni*, Vaccine, MHC Class I, MHC Class II, T-cell epitopes, B-cell epitopes, Guillain Barré syndrome

## Abstract

**Purpose:**

*Campylobacter jejuni* is the one of the leading causes of bacterial diarrheal illness worldwide. This study aims to design specific epitopes for the utility of designing peptide vaccine(s) against *C. jejuni* by targeting invasive, virulent and membrane associated proteins like FlaA, Cia, CadF, PEB1, PEB3 and MOMP.

**Methods:**

In the present study, various immunoinformatics approaches have been applied to design a potential epitope based vaccine against *C. jejuni*. The tools include Bepipred, ABCpred, Immune Epitope databse (IEDB) resource portal, Autodock vina etc.

**Results:**

Peptides “EINKN”, “TGSRLN”, “KSNPDI”, “LDENGCE” respectively from FlaA, MOMP, PEB3, CadF proteins were found to be the most potential B cell epitopes while peptides “FRINTNVAA”, “NYFEGNLDM”, “YKYSPKLNF”, “YQDAIGLLV”, “FRNNIVAFV” and “LIMPVFHEL” respectively from Fla, CadF, MOMP, PEB1A, PEB3 and Cia might elicit cell mediated immunity and “IFYTTGSRL” from MOMP protein might elicit both humoral and cell-mediated immunity. All these potential peptidic epitopes showed almost 80–100 % conservancy in different strains of *C jejuni* with varying proportions of population coverage ranging from 22–60 %. Further authentication of these peptide epitopes as probable vaccine candidate was mediated by their binding to specific HLA alleles using *in silico* docking technique.

**Conclusion:**

Based on the present study, it could be concluded that these predicted epitopes might be used to design a vaccine against *C. jejuni* bacteria and thus, could be validated in model hosts to verify their efficacy as vaccine.

**Electronic supplementary material:**

The online version of this article (doi:10.1186/s40203-016-0020-y) contains supplementary material, which is available to authorized users.

## Background

*Campylobacter jejuni*, a gram-negative bacillus, is considered to be a commensal organism of chicken gut. It causes gastroenteritis in humans (Nyati and Nyati, 2013) that can ultimately lead to Guillain Barré syndrome (GBS). GBS remains one of the most fascinating yet challenging conditions despite considerable advances in its understanding and treatment over the past 10 years have been put together (Winer, 2001). It is likely that immune responses directed against infecting organisms are involved in the pathogenesis of GBS by cross-reaction with neural tissues. The infecting organism induces humoral and cellular immune responses that, because of the sharing of homologous epitopes (molecular mimicry), cross-react with ganglioside surface components of peripheral nerves (Hahn, 1998). It may happen at any age and both sexes have equal possibilities to be predisposed to this disease (NIH, 2015).

The reported incidence of the GBS in Western countries in 2011 ranges from 0.89 to 1.89 cases per 100,000 person/years although an increase of 20 % is seen with every 10-year rise in age after the first decade of life (Sejvar et al., 2011). In Bangladesh, non-polio acute flaccid paralysis (AFP) cases are frequently diagnosed though poliomyelitis has been eradicated from the country (Sejvar et al., 2011). In 2006 and 2007, a total of 1619 and 1844 AFP cases, respectively, were reported in children under 15 years of age, of which 37 % and 43 % cases, respectively, were identified as GBS case. Overall, the crude incidence rate of GBS in children <15 years of age varied from 1.5 to 2.5 cases per 100,000 population per year in the 6 divisions of Bangladesh.

*Campylobacter jejuni* has become the most frequent antecedent pathogen for GBS (Hahn, 1998). The pathogenic clinical strain NCTC11168 was the first *C. jejuni* strain to be sequenced and has been a widely used laboratory model for studying its pathogenesis (Parkhill et al., 2000) and in this study this strain was taken into consideration for designing epitopes. The mechanism of *C. jejuni-*mediated enteritis is proposed to be multifactorial. Following ingestion, *C. jejuni* infect and invade the epithelium of the small intestine and colon which depends on motility mediated by polar flagella and outer membrane adhesins, including PEB1a, PEB3, MOMP and CadF. Thus, surface-exposed bacterial ligands play major roles in mediating mucosal adhesion and invasion (Mahdavi et al., 2014) and these host–pathogen interfaces during *C. jejuni* infection are complex, vibrant and involved in the nicking of host cell environment, enzymes and pathways (Ingale and Goto, 2014).

These proteins are particularly important for vaccine development as they mediate pathogen entry and colonization and are also the primary target of adaptive immune response. Well characterized protective epitopes designed from these proteins can be a great help for offering consistent, cost effective and quality therapeutics over the current treatment. In the present study, *in silico* drug designing and immunoinformatics strategies have been exploited using bioinformatics software. Epitope-based immunoinformatics study was carried out for these six proteins of *C. jejuni* in order to predict informative epitopes which can be helpful for future vaccine development.

## Methods

**Fig. 1:**
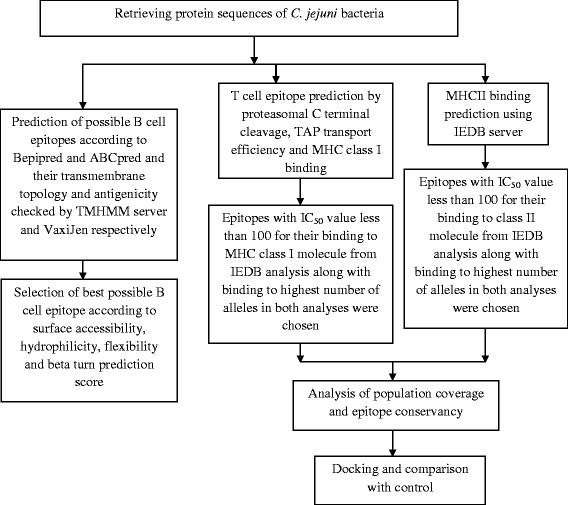
Flowchart displaying the protocols employed to predict B cell and T cell epitopes

To identify the best probable B- and T-cell peptides which could be used to design an effective vaccine, different approaches were taken into consideration in this study and an outline of the methodology has been depicted in Fig. 1.

### Retrieval of protein sequences

Sequences of flaA, CadF, Cia, PEB1, PEB3 and MOMP of strain 11168 of *C. jejuni* were retrieved from uniprot (www.uniprot.org) in FASTA format.

### Prediction of putative B cell epitopes, their antigenicity and transmembrane properties

The whole protein sequences were analyzed for B cell epitope prediction. In order to predict linear B-cell epitopes, Bepipred tool (Larsen et al., 2006) with default threshold value 0.35 was employed. For cross checking the predicted epitope(s), the protein sequences were also subjected to ABCpred server (www.imtech.res.in/raghava/abcpred/) (Saha and Raghava, 2006) by setting cut-off value at 0.51 and the length of the epitopes was set to be 16 mer. ABCpred generates datasets of fixed length patterns by eliminating or adding residues at the terminal ends of the peptides.

Antigenicity and transmembrane topology of the peptide sequences were also checked by VaxiJen V2.0 server (http://www.ddg-pharmfac.net/vaxijen/VaxiJen/VaxiJen.html) with 0.5 as threshold (Doytchinova and Flower, 2007) and TMHMM v0.2 server (Krogh et al., 2001), respectively.

#### Prediction of surface accessibility, hydrophilicity, flexibility and beta-turn of the predicted epitopes

A B­cell epitope is characterized by its antigenicity, hydrophilicity, accessibility and flexibility (Fieser et al., 1987). Therefore, Emini surface accessibility prediction tool (Emini et al., 1985), Parker hydrophilicity scale (Parker et al., 1986), Karplus and Schulz flexibility scale (Karplus and Schulz, 1985) and Chou and Fashman beta-turn prediction tool (Chou and Fasman, 1978) all with default parameters were applied to predict the surface exposure probabilities, hydrophilicity, flexibility and beta turn of the amino acids within the predicted epitopes respectively. The results from these analyses were cross-referenced and apparently common findings were taken as the most probable B-cell epitopes.

### T cell epitope prediction

T cell epitope was predicted by tools available in Immune Epitope Database (IEDB) (tools.immuneepitope.org) which provides a catalog of experimentally characterized B and T cell epitopes, as well as data on Major Histocompatibility Complex (MHC) binding and MHC ligand elution experiments (Vita et al., 2010).

#### Proteasomal cleavage, TAP, MHC I binding prediction

A combined algorithm of MHC­1 binding, transporter of antigenic peptide (TAP) transport efficiency and proteasomal cleavage efficiency was involved to predict overall scores for each peptide's intrinsic potential of being a T cell epitope. The Stabilized Matrix Base Method (SMM) was used to calculate IC_50_ values of peptide (obtained from whole protein) binding to MHC­1 molecules. For all the alleles, peptide length was set to 9 amino acids prior to the prediction. The parameters for detection of immunogenicity (TAP score, proteasomal score, and IC_50_ values) were normalized on a scale of 0 to 1 and were given a weighted score to prioritize the parameters (Sakib et al., 2014). The alleles having binding affinity IC_50_ less than 100 nm were chosen for further consideration.

#### MHC class I and class II binding prediction

The nonamers were given as input in MHC class l binding prediction tool available in the Immune Epitope Database (IEDB) server (http://tools.immuneepitope.org/mhci/). The peptides which interacted with highest number of alleles were selected.

On the other hand, the whole protein sequences were submitted in the IEDB MHC class II binding prediction tool (http://tools.immuneepitope.org/mhcii/) since MHC class II can accommodate much longer peptides – possibly even whole proteins. The Stabilized Matrix Base Method (SMM) was used to calculate IC_50_ values of peptide binding to MHC­ll molecules. The peptides (containing 15 amino acid residues) that interacted with highest number of alleles were again selected.

In both cases, SMM-align method was employed to find out good binders and the cut-off value of IC_50_ was set 100 nM. The overlapping epitopes between MHC I and MHC II binding predictions which interacted with highest number of alleles (minimum 3 alleles) were finally selected to predict epitope conservancy and population coverage.

### Analysis of population coverage by the predicted epitopic peptides

To become a good vaccine candidate, prediction of T cell epitope is not enough. The predicted peptide(s) should effectively cover human population. To find out the population coverage of the individual epitopes, predicted epitopic sequences with the corresponding Class l HLA alleles were submitted to the population coverage analysis tool of IEDB (http://tools.immuneepitope.org/tools/population/iedb_input) by maintaining the default analysis parameters. This server employs the most comprehensive database allelefrequencies.net for their coverage calculation.

### Analysis of conservancy, variability and allergenicity of the predicted epitopes

The epitopes were subjected to analyze for comparing conservancy among different strains retrieved from different countries of the world using IEDB analysis tool. Besides, Allerdictor tool was employed to find out whether these proposed epitopes showed any kind of allergenicity (Dang and Lawrence, 2014). It is a fast and accurate sequence-based allergen prediction tool that models protein sequences as text documents and employs support vector machine for allergen prediction. Their variability was also determined using Protein Variability server (Garcia-Boronat et al., 2008).

### T cell epitope prediction from conserved region analysis

To find the conserved region, retrieved sequences of each protein were aligned using ClustalW tool that uses Gonnet matrix in MEGA software (v 5.2) along with 1000 bootstrap value and default parameters. The aligned sequences from each protein that generated the highest number of identical amino acid with no gap were considered as the conserved regions for respective proteins. In the present study, the minimum length of the conserved regions was set at 15 as binding predictions for MHC class II alleles use to create 15mers. The identified largest conserved regions were further analyzed using similar steps as done in case of full protein sequence analysis.

### Docking of the best selected peptides in the binding groove of HLA alleles

“PEPFOLD” server was used to predict the three dimensional structure of selected peptide “FRLSDSLAL” and “NYFEGNLDM” from CadF (Maupetit et al., 2009; Thevenet et al., 2012). The best models provided by the server were selected for the docking study.

On the other hand, the three dimensional structure of the HLA-B*27:05 allele, as it was found to interact with “FRLSDSLAL” peptide, was retrieved from protein data bank (PDBID: 1JGE) which was deposited as a complex with nonamer peptide m9. M9 is a model synthetic peptide that has the sequence of (GRFAAAIAK) (Hülsmeyer et al., 2002). After visualizing the structures in PyMol molecular graphics system, the peptide sequence was removed from the binding groove of the MHC class I allele. The AutoDOCK tool from the MGL software package (version 1.5.6) was employed for the docking purpose (Morris et al., 1998; Morris et al., 2009). The predicted 3D structure of HLA-C*07:02 allele was obtained from a previous study (Sakib et al., 2014).

These structures were then further prepared for running docking in AutoDock tool by adding polar hydrogens and water molecules. For the docking study both the HLA allele and ligand files were converted into PDBQT format. In case of HLA-B*27:05, the grid/space box center was set at 22.241, 15.002 and 21.475 Å in the *x*-, *y*-, and *z*-axes, respectively to allow the epitope “FRLSDSLAL” to bind to the binding groove of the allele. The size was set at 52, 34 and 30 Å in the *x*, *y*, and *z* dimensions, respectively. All the analyses were done at 1.00 Å spacing. The thoroughness of global search algorithm i.e. the exhaustiveness parameter was kept at 8.00, while the number of outputs was set at 10. The docking was conducted using AutoDOCK Vina program based on the set parameters. The PDBQT files were converted in PDB format using OpenBabel (version 2.3.1) and visualized in PyMOL molecular Graphics system. The same procedure was also followed for the binding of “NYFEGNLDM” nonamer to HLA-C*07:02 allele. In that case, the grid/space box center was set at -13.677, 2.561 and 30.904 Å in the *x*-, *y*- and *z*-axes respectively to allow the epitope to bind to the groove of the HLA-C*07:02. The size was set at 30, 46 and 50 Å in the *x*, *y*, and *z* dimensions, respectively.

The best output was selected on the basis of the best binding energy. To compare the binding of the predicted folded structure of the nonamer (“FRLSDSLAL”) to the binding groove of the class I MHC allele, the removed nonamer m9 was also allowed to dock as control. The control nonamer was docked by considering similar parameters.

The 3D structure of MHC class I H-2Kb molecule complexed with octapeptide PKB1 (“KVITFIDL”) was retrieved from Protein Data Bank Database (ID: IKJ3) and visualized using PyMOL Graphics. The octapeptide was excluded before applying the structure of H-2Kb for comparing the validated data obtained for predicted structure of HLA-C*07:02.

Also, to assess HLA-C*07:02-epitope docking results, octapeptide PKB1 (“KVITFIDL”) was used as the control. This peptide was docked with HLAs, HLA-C*07:02, and H-2Kb. The test epitope(s) and the control peptide were docked by setting similar parameters for each trial and successful binding of this peptide to these HLAs was demonstrated. Finally, H-2Kb - KVITFIDL docking result was used as control to compare with the test docking results of HLA-C*07:02 complexed with selected epitopes.

## Results

### Identification of B cell epitopes

After retrieving the sequences of FlaA, PEB1a, PEB3, MOMP, CadF and Cia proteins of *Campylobacter jejuni* strain 11168 several epitopes were predicted. However, only those peptides that were found fully overlapping between Bepipred and ABCpred prediction tools (Larsen et al., 2006; Saha and Raghava, 2006) were selected for further analysis by VaxiJen and TMHMM server (Doytchinova and Flower, 2007; Krogh et al., 2001). On the basis of VaxiJen scores and transmembrane topology, epitopes “TGLGALADEINKNADK”, “DWSKSNPDIGTAVAIE”, “GEEIFYTTGSRLNGDT” and “PREGALLDENGCEKTI” respectively from FlaA, PEB3, MOMP and CadF proteins were found to be antigenic (VaxiJen score ≥ 0.5) and they also fulfilled the criteria of exomembrane characteristics (Table 1).

**Table 1:** Predicted B cell epitopes of FlaA, PEB3, MOMP, CadF and Cia protein from *Campylobacter jeuni* and their VaxiJen score, transmembrane topology and position

Protein	Predicted B cell epitope	VexiJen Score (Threshold > 0.5)	Prediction of transmembrane helix using TMHMM server	Position of the selected sequences
FlaA	TGLGALADEINKNADK	0.6933	Outside	212–227
PEB3	DWSKSNPDIGTAVAIE	0.6732	Outside	191–206
MOMP	GEEIFYTTGSRLNGDT	0.7115	Outside	318–333
CadF	PREGALLDENGCEKTI	0.8825	Outside	195–210

By using different tools in IEDB, it was found linear peptides “EINKN”, “TGSRLN”, “KSNPDI”, “LDENGCE” respectively from FlaA, MOMP, PEB3, CadF proteins fulfilled the criteria of surface accessibility, hydrophilicity, flexibility and beta-turn for becoming the most probable B cell epitope (Additional file 1: Table S1).

### Prediction of T cell epitopes

#### MHC class I and class II epitope identification

Analysis using a combined algorithm integrating MHC class I binding, TAP efficiency and proteosomal cleavage prediction generated 562 processed T cell epitopes from FlaA, 250 processed T cell epitopes from PEB1, 241 epitopes from PEB3 protein, 414 from MOMP, 691 from CadF protein and 426 epitopes from Cia that interacted with different possible MHC I alleles with the IC50 value <100 nM.

These processed peptides were then analyzed by SMM based IEDB MHC I prediction tool and this method retrieved 59 nonamers from flaA, 49 nonamers from MOMP, 18 from PEB1 protein, 18 from PEB3, 32 from CadF protein and 57 from Cia with the IC_50_ value <100 nM. A good epitope should also interact with as many as MHC alleles. Thus, among the total peptides, only those peptides which interacted with minimum four MHC class I alleles as well as were found to be the core sequences of 15mer MHC class II alleles, were selected from each protein (Additional file 1: Table S2, Table S3, Table S4, Table S5, Table S6 and Table S7).

#### Population coverage, epitope conservancy, variability and allergenicity analysis

Over a thousand different human MHC (HLA) alleles are known and different HLA types are expressed at different frequencies in different ethnicities. Population coverage by the most probable epitopes varied between 20 % – 65 % when MHC class I alleles were considered (Additional file 1: Table S2, Table S3, Table S4, Table S5, Table S6 and Table S7).

The epitopes which were found to have large world population coverage were selected as most probable epitopes for vaccine design (Table 3). From FlaA “FRINTNVAA” peptide, from PEB3 protein “FRNNIVAFV” epitope, from MOMP protein “IFYTTGSRL” and “YKYSPKLNF”, from CadF protein “NYFEGNLDM”, from PEB1A “YQDAIGLLV” and from Cia protein “LIMPVFHEL” epitope were selected as best potential vaccine candidates. The regions where these epitopes showed comparatively higher population coverage is presented graphically (Additional file 1: Figure S1).

All of the epitopes were found to be non allergen (Table 2). The protein variability index of these epitopes was also calculated (Additional file 1: Figure S2).

**Table 2:** Most probable predicted epitopes interacting with different MHC class I and class II alleles from IEDB analysis tool

Proteins used	Predicted peptides using IEDB tool	Interacting MHC-I alleles	Population coverage	Interacting MHC-II alleles	Conservancy	Allergenicity
FlaA	FRINTNVAA	HLA-C*03:03 HLA-C*12:03 HLA-C*14:02 HLA-B*39:01 HLA-C*07:01 HLA-C*06:02	51.40 %	HLA-DRB1*04:01 HLA-DRB1*13:02 HLA-DRB1*01:01	100 % conserved among 23 strains, 88.89 % conserved in 4 strains	Non-allergen
CadF	NYFEGNLDM	HLA-C*14:02 HLA-C*07:01 HLA-C*06:02 HLA-C*12:03 HLA-C*07:02 HLA-B*15:02	60.72 %	HLA-DRB1*04:05	100 % conserved among 13 strains	Non-allergen
MOMP	IFYTTGSRL	HLA-C*03:03 HLA-C*14:02 HLA-B*15:02 HLA-C*12:03	22.69 %	HLA-DRB1*07:01 HLA-DRB1*01:01 HLA-DPA1*02:01 HLA-DPB1*01:01	100 % conserved among 18 strains, 77.78 % conserved among 5 strains	Non-allergen
	YKYSPKLNF	HLA-C*03:03 HLA-B*15:02 HLA-C*14:02 HLA-C*12:03	22.69 %	HLA-DRB1*11:01 HLA-DRB1*07:01	100 % conserved among 18 strains	Non-allergen
PEB1A	YQDAIGLLV	HLA-A*02:06 HLA-C*12:03 HLA-C*05:01 HLA-A*02:01 HLA-C*08:02	53.42 %	HLA-DRB1*01:01 HLA- DRB1*07:01	100 % conserved among 8 strains, 33.33 % conserved among 10 strains, 22.22 % conserved in 1 strain	Non-allergen
PEB3	FRNNIVAFV	HLA-C*06:02 HLA-C*12:03 HLA-C*07:01 HLA-C*05:01	47.63 %	HLA- DRB1*13:02 HLA- DRB1*04:04 HLA- DRB1*01:01 HLA- DRB3*01:01	100 % conserved among 40 strains, 88.89 % conserved in 1 strain	Non-allergen
Cia	LIMPVFHEL	HLA-A*02:06 HLA-A*02:01 HLA-C*15:02 HLA-C*12:03 HLA-A*68:02	50.86 %	HLA-DPA1*02:01 HLA-DPB1*01:01 HLA-DPA1*01:03 HLA-DPB1*02:01	100 % conserved among 20 strains	

The conservancy analyses revealed that nonamers “NYFEGNLDM” from CadF, “LIMPVFHEL” from Cia and “YKYSPKLNF” from MOMP protein were 100 % conserved among all strains analyzed while the other candidate peptides varied between 33.33 % to 100 % conservancy (Table 2). In addition, “NYFEGNLDM” from CadF demonstrated highest world population coverage which was 60.72 %.

It was also found out “YYQDAIGLL” and “MVFRKSLLK” from PEB1A; “YRTFNVLAK” from PEB3 and “FALKGSIEV” from MOMP proteins showed large population coverage, interacted with 6-9 MHC class I alleles and also were found overlapping with 15mer peptides of MHC class II alleles. The nonamers “YYQDAIGLL”, “MVFRKSLLK”, “YRTFNVLAK” and “FALKGSIEV” showed 55.69 %, 56.39 %, 54.57 % and 55.25 % world population coverage, respectively.

### Multiple sequence alignment and prediction of T cell epitopes from conserved regions of each protein

In another approach, the sequences of the six proteins from different isolates of *Campylobacter jejuni* were retrieved from NCBI GenBank sequence database (www.ncbi.nlm.nih.gov/genbank/) and UniProt (www.uniprot.org/). The strains included 81-176; ATCC_700819/NCTC_11168; ATCC_BAA-1458/RM4099/269.97; 260.94; jejuni_CG8421; CF93-6; jejuni_IA3902; RM1221; CJM1cam; jejuni_M1; NCTC_11168-BN148 and jejuni_81116. The retrieved sequences were then aligned using ClustalW tool that uses Gonnet matrix in MEGA software (v 5.2). The conserved regions from each protein with the highest length were then used for T cell epitope prediction. They were subjected to IEDB and SYFPEITHI MHC class I and class II binding prediction tool. It was found out “FRLSDSLAL” epitope from CadF protein which stood out as the best potential vaccine candidate from that protein (Additional file 1: Figure S3) was also common among the most probable epitopes from CadF during whole protein sequence analysis. The epitope showed 37.98 % population coverage and as it came from a conserved region its conservancy among the different isolates was 100 % (Table 3).

**Table 3:** Predicted epitope from CadF protein found common in both whole protein analysis and conserved region analysis

Protein	Peptide	Interacting MHC-I alleles	Population Coverage	Overlapping 15 mer peptides	Interacting MHC-II alleles	Conservancy	Allergenicity
CadF	FRLSDSLAL	HLA-B*39:01 HLA-C*03:03 HLA-B*15:02 HLA-C*14:02 HLA-C*07:02 HLA-B*27:05 HLA-B*27:09	37.98 %	GVKFRLSDSLALRLE	HLA-DRB1*07:01 HLA-DRB1*01:01 HLA-DRB5*01:01 HLA-DRB1*09:01 HLA-DRB1*04:01 HLA-DRB1*0101 HLA-DRB1*0301 HLA-DRB1*1501	100 %	Non-allergen
				GAGVKFRLSDSLALR	HLA-DRB1*07:01 HLA-DRB1*01:01 HLA-DRB5*01:01 HLA-DRB1*09:01 HLA-DRB1*04:01 HLA-DRB1*0301		Non-allergen
				KFRLSDSLALRLETR	HLA-DRB1*07:01 HLA-DRB1*01:01 HLA-DRB1*09:01 HLA-DRB5*01:01 HLA-DRB1*0301 HLA-DRB1*1501		Non-allergen

### Docking

**Fig. 2:**
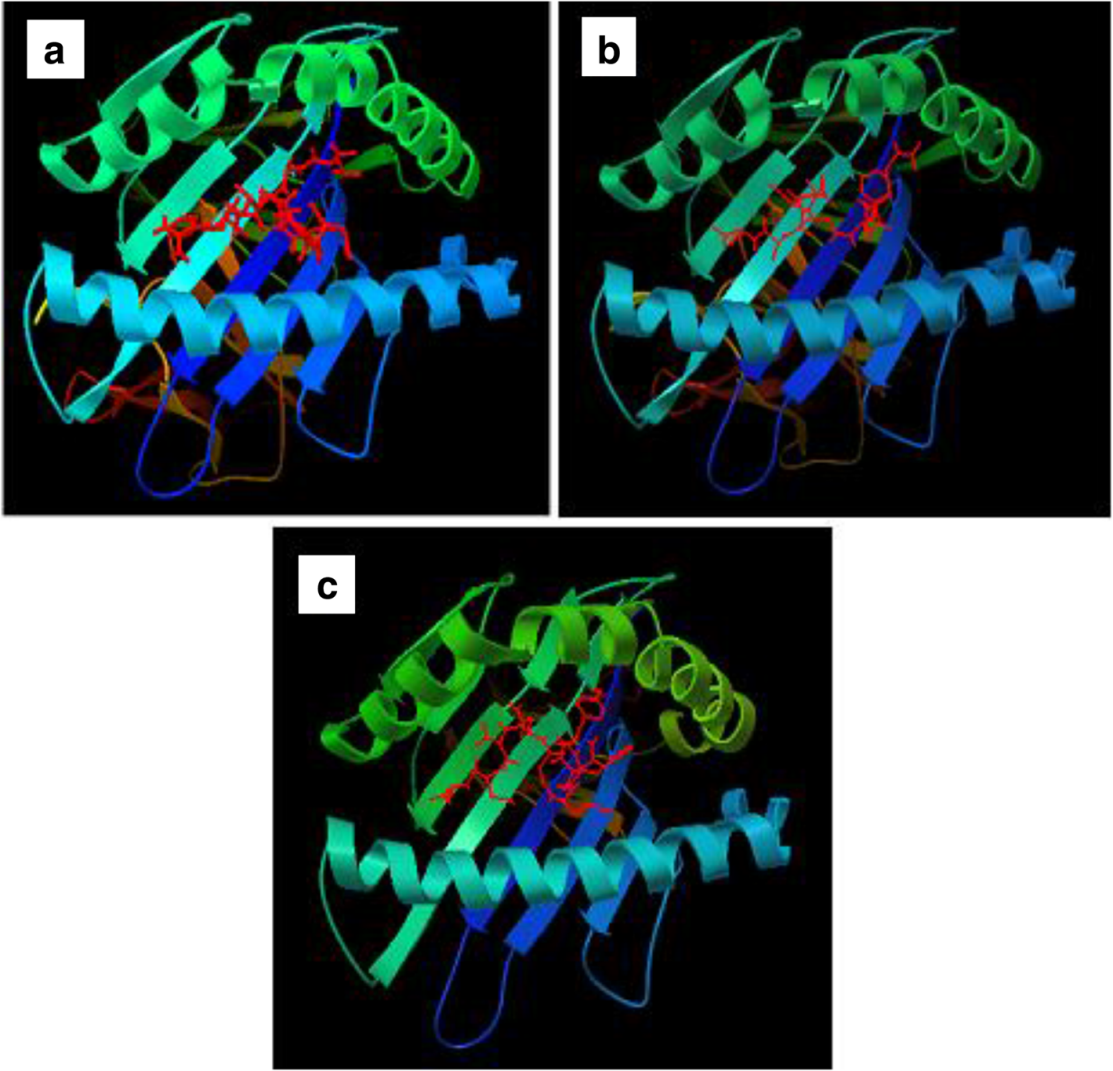
Docking to predict the binding of predicted and control epitopic peptides to MHC class I molecule, HLA-B*27:05. The bindings of 3D structures of **a** predicted peptide, “FRLSDSLAL”; **b** the control peptide, “m9” to the binding grooves of HLA-B*27:05 and binding energies were found to be almost similar (-6.1 and -6.7 kcal/mol, respectively) and **c** Docking to predict the binding of predicted epitopic peptide “NYFEGNLDM” to MHC class I molecule, HLA-C*07:02. Docking of control peptide to the HLA-C*07:02 groove was done in previous study (Sakib et al*.*, 2014)

Binding models of the best probable epitopes (“FRLSDSLAL” and “NYFEGNLDM” from CadF) to its specific HLA molecules were observed using AutoDock Vina. Epitope “FRLSDSLAL” bound to the binding groove of HLA-B*27:05 with the binding energy -6.1 kcal/mol and “NYFEGNLDM” bound with HLA-C*07:02 with the binding energy -6.3 kcal/mol (Fig. 2a and c). On the other hand after setting same parameters, the binding energy of the control peptides “m9” to the binding grooves of class I MHC allele- HLA-B*27:05 were estimated to be -6.7 kcal/mol (Fig. 2b).

## Discussion

*Campylobacter jejuni* is recognized as a major risk factor for the onset of Guillain‐Barré syndrome, which is a serious post‐infection complication characterized by acute and progressive neuromuscular paralysis (Allos, 2001). Almost 25 %–40 % of GBS patients worldwide suffer from *C. jejuni* infection 1–3 weeks prior to the illness (Mishu and Blaser, 1993). Up to 20 % of GBS patients remain severely disabled and approximately 5 % die in the western countries (Yuki, 2012). *C. jejuni* is also responsible for gastroenteritis (Gblossi Bernadette et al., 2012) and a diarrheal disease called campylobacteriosis which is considered as a major health issue attributable to unavailability of appropriate vaccines and clinical treatment options. Existing therapies are trusted only on a much smaller number of drugs, most of them are insufficient because of their severe host toxicity or drug-resistance phenomena (Ingale and Goto, 2014). Particularly the increasing prevalence of drug‐resistant *Campylobacter* has compromised the effectiveness of the currently used antibiotics and poses a significant threat to public health in many countries (Blaser and Engberg, 20082008). Moreover, treatment of GBS caused by this organism is highly expensive (Nagpal et al., 1999). Thus, an effective cost effective prevention scheme would be most desirable for the public health providers as well as the scientific communities.

To find out remedial alternatives, the identification of new biotargets is therefore highly anticipated. Understanding the molecules involved in pathogenesis has the potential to yield new and exciting strategies for therapeutic intervention (Ingale and Goto, 2014). The flagellum (FlaA, out of two flagellar proteins), outer membrane adhesions- major outer membrane protein (MOMP), Campylobacter adhesion to fibronectin (CadF), Campylobacter invasion antigen (Cia), the major cell binding factor PEB1 and the major antigenic peptide PEB3 of C. jejuni facilitate its colonization into the intestinal tract of animals (Day et al., 2009; Mahdavi et al., 2014). Therefore, in this study, we made an attempt to design epitope based vaccines from these proteins which could be tested for their efficacy in eliciting immunity through humoral and cell mediated immune responses. The ultimate goal of epitope prediction is to aid the design of molecules that can mimic the structure and function of a genuine epitope and replace it in vaccine design (Gomara and Haro, 2007; Peters et al., 2005). However, the activation of cytotoxic T-cells requires recognition of specific peptides bound to MHC class I molecules (Sette et al., 2001). And while sequences from pathogens provide a huge amount of potential vaccine candidates, it is estimated that only one in 100 to 200 peptides actually binds to a particular MHC (Yewdell and Bennink, 1999). Therefore, a good computational prediction method could significantly reduce the number of peptides that have to be synthesized and tested. With the advent of computers and informatics, new approaches have been devised that facilitate immunoinformatics which targets the use of mathematical and computational approaches to predict T-cell and B-cell immune epitopes (De Groot et al., 2002).

The identification of B-cell epitopes is rather important to immunodetection and immunotherapeutic applications since an epitope as the minimal immune unit is strong enough to elicit a potent humoral immune response with no harmful side effects to human body (Sun et al., 2013).

In this study, linear B-cell epitopes were chosen with two different algorithms- BepiPred and ABCpred. Only overlapping peptides which were chosen by both algorithms as well as satisfied the scores of VaxiJen and passed transmembrane topology were selected as potential B-cell epitopes and subjected to further analysis using the parameters of surface accessibility, hydrophilicity, flexibility, and beta-turn. By cross-referencing all the data, we predicted that the peptide sequences “EINKN”, “TGSRLN”, “KSNPDI”, “LDENGCE” respectively from FlaA, MOMP, PEB3, CadF proteins were capable of inducing the desired immune response as B cell epitope. Among them “EINKN” and “LDENGCE” were also found non-allergen as predicted by AllerTOP 1.0 tool.

For T cell epitope prediction, plenty of algorithms are freely available and in this study we employed IEDB analysis tool which is possibly the most wide-ranging database offering several B cell and T cell epitope-related analysis and prediction tools as well as provides both intrinsic biochemical and extrinsic context dependent information about them (Zhang et al., 2008). Initial analysis of the data showed that among all the predicted epitopes, “FRINTNVAA” peptide from FlaA, “FRNNIVAFV” epitope from PEB3, “IFYTTGSRL” and “YKYSPKLNF” of MOMP, from CadF “NYFEGNLDM”, from PEB1A “YQDAIGLLV” and from Cia protein “LIMPVFHEL” epitopes interacted with the highest numbers of MHC class I alleles, were the core peptides of a good number of MHC class II binding predictions and also demonstrated large world population coverage. They were also found to be non–allergen from AllerTop 1.0 analysis. Further analysis found out “YYQDAIGLL” and “MVFRKSLLK” from PEB1A protein; “YRTFNVLAK” from PEB3 and “FALKGSIEV” from MOMP protein showed highest population coverage, interacted with 6-9 MHC class I alleles and also were found to be overlapping with a good number of 15mer peptides of MHC class II alleles. All the epitopes showed 100 % conservancy among majority of the strains.

In another approach, all the available strains from each protein were aligned and the largest conserved regions found from each alignment were subjected to T cell epitope prediction by both IEDB and SYFPEITHI analysis tools with the aim to propose the most probable epitope(s) that could be used to design a vaccine that would be universally applicable. Interestingly, “FRLSDSLAL” epitope was revealed as the best potential vaccine candidate from CadF protein which was also common among the most probable epitopes from CadF during whole protein sequence analysis. The epitope showed 37.98 % population coverage. Another important finding was that the most probable predicted B cell epitope from MOMP protein “TGSRLN” peptide overlapped with the predicted T cell epitope “IFYTTGSRL” in this study. So this epitope based vaccine would be able to elicit both humoral and cell mediated immunity.

## Conclusion

In this study, we made an attempt to design epitope based vaccines against *Campylobacter jejuni* which could be tested for their efficacy in eliciting immunity through humoral and cell mediated immune responses. The results of our study provide computational data for the identification and screening of epitopes, and may be used for the development of epitope vaccines that have an enhanced safety and efficacy. This may result in the provision of improved regimens for the prevention of GBS. Our findings are based on sequence analysis and computational predictions. However, to prove the effectiveness of mounting an immune response, both *in vitro* and *in vivo* studies are required along with this *in silico* study.

## Additional file

Additional file 1: Table S1. Accessibility, hydrophilicity, flexibility and beta- turn prediction score for each residue of the predicted common linear B cell epitopes of FlaA, MOMP, PEB3 and CadF protein. **Table S2:** Most probable predicted epitopes of FlaA protein interacting with different MHC class I and class II alleles from IEDB analysis tool. **Table S3:** Most probable predicted epitopes of MOMP protein interacting with different MHC class I and class II alleles from IEDB analysis tool. **Table S4:** Most probable predicted epitopes of PEB1A protein interacting with different MHC class I and class II alleles from IEDB analysis tool. **Table S5:** Most probable predicted epitopes of PEB3 protein interacting with different MHC class I and class II alleles from IEDB analysis tool. **Table S6:** Most probable predicted epitopes of CadF protein interacting with different MHC class I and class II alleles from IEDB analysis tool. **Table S7:** Most probable predicted epitopes of Cia protein interacting with different MHC class I and class II alleles from IEDB analysis tool. **Figure S1:** Population coverage by MHC Class I restricted epitopes predicted from FlaA, CAdF, MOMP, PEB1A, PEB3, Cia proteins of *Campylobacter jejuni*. **Figure S2:** The protein variability index of the epitopes from a) FlaA, b) PEB1A, c) PEB3, d) MOMP, e) Cia and f) CadF protein was determined by using PVS server. The epitope positions are indicated by arrow signs. The conservancy threshold was 1.0 in this analysis. X axis indicates the amino acid position in sequences and Y axis indicates the Shannon variability. **Figure S3:** Multiple Sequence Alignment of CadF protein done by CLUSTAL. The conserved epitope sequence is shown in yellow. (DOC 382 kb)
