# Supplementary material for: In silico proposition to predict cluster of B- and T-cell epitopes for the usefulness of vaccine design from invasive, virulent and membrane associated proteins of C. jejuni
Source: In Silico Pharmacol. 2016 Jul 4;4:5. doi: 10.1186/s40203-016-0020-y (PMC4932005; doi:10.1186/s40203-016-0020-y)
Supplement: Additional file 1: Table S1. — Accessibility, hydrophilicity, flexibility and beta- turn prediction score for each residue of the predicted common linear B cell epitopes of FlaA, MOMP, PEB3 and CadF protein. Table S2: Most probable predicted epitopes of FlaA protein interacting with different MHC class I and class II alleles from IEDB analysis tool. Table S3: Most probable predicted epitopes of MOMP protein interacting with different MHC class I and class II alleles from IEDB analysis tool. Table S4: Most probable predicted epitopes of PEB1A protein interacting with different MHC class I and class II alleles from IEDB analysis tool. Table S5: Most probable predicted epitopes of PEB3 protein interacting with different MHC class I and class II alleles from IEDB analysis tool. Table S6: Most probable predicted epitopes of CadF protein interacting with different MHC class I and class II alleles from IEDB analysis tool. Table S7: Most probable predicted epitopes of Cia protein interacting with different MHC class I and class II alleles from IEDB analysis tool. Figure S1: Population coverage by MHC Class I restricted epitopes predicted from FlaA, CAdF, MOMP, PEB1A, PEB3, Cia proteins of Campylobacter jejuni. Figure S2: The protein variability index of the epitopes from a) FlaA, b) PEB1A, c) PEB3, d) MOMP, e) Cia and f) CadF protein was determined by using PVS server. The epitope positions are indicated by arrow signs. The conservancy threshold was 1.0 in this analysis. X axis indicates the amino acid position in sequences and Y axis indicates the Shannon variability. Figure S3: Multiple Sequence Alignment of CadF protein done by CLUSTAL. The conserved epitope sequence is shown in yellow. (DOC 382 kb) [file 40203_2016_20_MOESM1_ESM.doc]

Table S1: Accessibility, hydrophilicity, flexibility and beta- turn prediction score for each residue of the predicted common linear B cell epitopes of FlaA, MOMP, PEB3 and CadF protein

| Protein | Peptide | Emini Surface accessibility score for each residue  (using default parameters) | | Parker Hydrophilicity Prediction score for each residue (using default parameters) | | Flexibility Prediction score for each residue (using default parameters) | | Beta-Turn Prediction score for each residue  (using default parameters) | |
| --- | --- | --- | --- | --- | --- | --- | --- | --- | --- |
| FlaA | TGLGALADEINKNADK | L | 0.189 |  |  |  |  |  |  |
|  |  | G | 0.132 | G | 0.343 | G | 0.961 | G | 0.94 |
|  |  | A | 0.223 | A | 1.029 | A | 0.955 | A | 1.011 |
|  |  | L | 0.468 | L | 1.329 | L | 0.962 | L | 0.894 |
|  |  | A | 0.332 | A | 1.5 | A | 0.981 | A | 0.877 |
|  |  | D | 0.528 | D | 1.686 | **D** | 1.006 | D | 0.877 |
|  |  | **E** | **1.28** | E | 2.2 | **E** | 1.021 | E | 0.927 |
|  |  | **I** | **2.037** | **I** | 4.514 | **I** | 1.028 | **I** | 1.066 |
|  |  | **N** | **1.232** | **N** | 4.514 | **N** | 1.033 | **N** | 1.066 |
|  |  | **K** | **1.188** | **K** | 4.514 | **K** | 1.039 | **K** | 1.066 |
|  |  | **N** | **3.391** | **N** | 4.214 | **N** | 0.961 | **N** | 1.104 |
| MOMP | GEEIFYTTGSRLNGDT | E | 0.629 |  |  |  |  |  |  |
|  |  | I | 0.918 | I | 1.057 | I | 0.979 | I | 0.887 |
|  |  | F | 0.765 | F | 0.986 | F | 0.966 | F | 0.801 |
|  |  | Y | 0.437 | Y | 0.686 | Y | 0.983 | Y | 0.919 |
|  |  | T | 0.835 | T | 0.5 | **T** | **1.024** | T | 1.017 |
|  |  | **T** | **1.89** | T | 2.243 | **T** | **1.063** | **T** | **1.086** |
|  |  | G | 0.995 | G | 2.243 | **G** | **1.084** | **G** | **1.084** |
|  |  | **S** | **1.108** | **S** | **3.514** | **S** | **1.083** | **S** | **1.144** |
|  |  | R | 0.76 | **R** | **3.586** | **R** | **1.064** | **R** | **1.23** |
|  |  | **L** | **1.282** | **L** | **4.271** | **L** | **1.048** | **L** | **1.301** |
|  |  | **N** | **1.381** | **N** | **4.2** |  |  | **N** | **1.216** |
| PEB3 | DWSKSNPDIGTAVAIE | **S** | **1.958** |  |  |  |  |  |  |
|  |  | **K** | **1.813** | **K** | **3.971** | **K** | **1.075** | **K** | **1.339** |
|  |  | **S** | **2.879** | **S** | **3.971** | **S** | **1.095** | **S** | **1.339** |
|  |  | **N** | **1.506** | **N** | **4.257** | **N** | **1.097** | **N** | **1.269** |
|  |  | P | 0.745 | **P** | **4.143** | **P** | **1.082** | **P** | **1.287** |
|  |  | D | 0.803 | **D** | **4.071** | **D** | **1.067** | **D** | **1.28** |
|  |  | I | 0.504 | **I** | **3.443** | **I** | **1.042** | **I** | **1.17** |
|  |  | G | 0.242 | G | 1.914 | **G** | **1.023** | G | 1.019 |
|  |  | T | 0.146 | T | 1.914 | T | 0.999 | T | 0.896 |
|  |  | A | 0.146 | A | -0.657 | A | 0.965 | A | 0.754 |
|  |  | V | 0.256 | V | 1.6 |  |  | V | 0.793 |
| CadF | PREGALLDENGCEKTI | **E** | **1.381** |  |  |  |  |  |  |
|  |  | G | 0.737 | G | 0.5 | **G** | **1.014** | G | 0.944 |
|  |  | A | 0.628 | A | 1.629 | A | 0.994 | A | 0.936 |
|  |  | L | 0.628 | L | 2.143 | L | 0.991 | L | 0.906 |
|  |  | **L** | **1.021** | L | 2.029 | **L** | **1.007** | L | 1.023 |
|  |  | **D** | **1** | D | 2.029 | **D** | **1.037** | D | 1.023 |
|  |  | E | 0.65 | E | 1.929 | **E** | **1.055** | **E** | **1.099** |
|  |  | **N** | **1.365** | **N** | **4.357** | **N** | **1.061** | **N** | **1.12** |
|  |  | **G** | **1.634** | **G** | **6.486** | **G** | **1.053** | **G** | **1.18** |
|  |  | **C** | **1.362** | **C** | **5.8** | **C** | **1.04** | **C** | **1.109** |
|  |  | E | 0.594 | **E** | **3.543** |  |  | **E** | **1.07** |

Table S2: Most probable predicted epitopes of FlaA protein interacting with different MHC class I and class II alleles from IEDB analysis tool

| Proteins used | Predicted peptides using IEDB tool | Interacting MHC-I alleles | Population Coverage | Predicted 15 mer peptides using IEDB tool | Core peptide | Interacting MHC-II alleles |
| --- | --- | --- | --- | --- | --- | --- |
| FlaA protein | FATMKTTAF | HLA-C*03:03  HLA-B*35:01  HLA-C*12:03  HLA-B*46:01  HLA-C*14:02 | 29.73% | STLSQFATMKTTAFG  TLSQFATMKTTAFGV  SQFATMKTTAFGVKD  LSQFATMKTTAFGVK QFATMKTTAFGVKDE FATMKTTAFGVKDET | FATMKTTAF | HLA-DRB1*01:01 |
|  |  |  |  | TLSQFATMKTTAFGV STLSQFATMKTTAFG SQFATMKTTAFGVKD LSQFATMKTTAFGVK |  | HLA-DRB1*04:01  HLA-DRB1*07:01 |
|  |  |  |  | LSQFATMKTTAFGVK |  | HLA-DRB5*01:01 |
|  | YAMAQANSV | HLA-C*12:03  HLA-C*15:02  HLA-C*14:02  HLA-A*02:06  HLA-A*02:03  HLA-A*68:02  HLA-C*03:03  HLA-A*02:02 | 29.13% | SGSYAMAQANSVQQN GSYAMAQANSVQQNV | YAMAQANSV | HLA- DQA1*05:01  HLA-DQB1*03:01 |
|  |  |  |  | AQSGSYAMAQANSVQ  QSGSYAMAQANSVQQ  SGSYAMAQANSVQQN  GSYAMAQANSVQQNV  SYAMAQANSVQQNVL YAMAQANSVQQNVLR |  | HLA-DRB1*01:01 |
|  |  |  |  | AQSGSYAMAQANSVQ  QSGSYAMAQANSVQQ  SGSYAMAQANSVQQNGSYAMAQANSVQQNV |  | HLA-DRB1*07:01  HLA-DRB1*09:01 |
|  | FSSGSGYSV | HLA-C*15:02  HLA-C*12:03  HLA-C*03:03  HLA-A*68:02  HLA-A*02:06  HLA-C*07:01 | 41.74% | SAGSGFSSGSGYSVG  AGSGFSSGSGYSVGS  SGFSSGSGYSVGSGK  GSGFSSGSGYSVGSG | FSSGSGYSV | HLA- DQA1*05:01  HLA-DQB1*03:01 |
|  |  |  |  | SAGSGFSSGSGYSVG GSGFSSGSGYSVGSG SGFSSGSGYSVGSGK |  | HLA-DRB1*01:01 |
|  |  |  |  | SAGSGFSSGSGYSVG  AGSGFSSGSGYSVGS  GSGFSSGSGYSVGSG  SGFSSGSGYSVGSGK |  | HLA-DRB1*07:01 |
|  | FRINTNVAA | HLA-C*03:03  HLA-C*12:03  HLA-C*14:02  HLA-B*39:01  HLA-C*07:01  HLA-C*06:02 | 51.40% | MGFRINTNVAALNAK | FRINTNVAA | HLA-DRB1*04:01 HLA-DRB1*13:02 HLA-DRB1*01:01 |
|  |  |  |  | GFRINTNVAALNAKA |  | HLA-DRB1*01:01 |
|  |  |  |  | FRINTNVAALNAKAN |  | HLA-DRB1*01:01 |
|  | FISQASVSL | HLA-C*03:03  HLA-B*15:02  HLA-C*12:03  HLA-A*02:06 | 21.57% | FGATQFISQASVSLR  GATQFISQASVSLRE  ATQFISQASVSLRES  TQFISQASVSLRESK  QFISQASVSLRESKG  FISQASVSLRESKGQ | FISQASVSL | HLA-DRB1*01:01 |
|  |  |  |  | FGATQFISQASVSLR  GATQFISQASVSLRE  ATQFISQASVSLRES TQFISQASVSLRESK |  | HLA-DRB1*07:01 |
|  |  |  |  | FGATQFISQASVSLR  GATQFISQASVSLRE  ATQFISQASVSLRES  TQFISQASVSLRESK |  | HLA-DRB5*01:01 |
|  |  |  |  | FGATQFISQASVSLR  GATQFISQASVSLRE  TQFISQASVSLRESK |  | HLA-DRB1*04:01 |
|  | IAISAASQL | HLA-C*03:03  HLA-C*12:03  HLA-B*15:02  HLA-C*15:02 | 23.90% | ANAIAISAASQLSTV  NAIAISAASQLSTVY  GFANAIAISAASQLS  FANAIAISAASQLST  AIAISAASQLSTVYN IAISAASQLSTVYNV | IAISAASQL | HLA-DRB1*07:01 |
|  |  |  |  | FANAIAISAASQLST ANAIAISAASQLSTV NAIAISAASQLSTVY |  | HLA-DRB1*01:01 |
|  | ETAGVTTLK | HLA-A*68:01  HLA-C*03:03  HLA-C*12:03  HLA-A*11:01  HLA-A*68:02 | 36.94% | KSLDASLSRLSSGLR LNSKSLDASLSRLSS SKSLDASLSRLSSGL NSKSLDASLSRLSSG | LDASLSRLS | HLA-DRB1*01:01 |
|  | TSFNGKQLL | HLA-C*07:01  HLA-C*06:02  HLA-B*15:02  HLA-C*12:03  HLA-C*03:03 | 49.12% | KSLDASLSRLSSGLR |  | HLA-DRB5*01:01 |
|  | DETAGVTTL | HLA-C*03:03  HLA-B*15:02  HLA-B*40:01  HLA-C*12:03  HLA-B*18:01 | 32.02% | KSLDASLSRLSSGLR SKSLDASLSRLSSGL LNSKSLDASLSRLSS NSKSLDASLSRLSSG |  | HLA-DRB1*09:01 |
|  |  |  |  | SKSLDASLSRLSSGL KSLDASLSRLSSGLR NSKSLDASLSRLSSG |  | HLA-DRB1*11:01 |
|  |  |  |  | SLDASLSRLSSGLRI |  | HLA-DRB1*07:01 |

Table S3: Most probable predicted epitopes of MOMP protein interacting with different MHC class I and class II alleles from IEDB analysis tool

| Protein used | Predicted peptides using IEDB tool | Interacting MHC-I alleles | Population Coverage | Predicted 15 mer peptides using IEDB tool | Core peptide | Interacting MHC-II alleles |
| --- | --- | --- | --- | --- | --- | --- |
| MOMP | FAVDSFMAA | HLA-C*03:03  HLA-C*12:03  HLA-A*02:06  HLA-B*35:01  HLA-B*15:02  HLA-C*14:02 | 30.66% | LAGEEIFYTTGSRLN AGEEIFYTTGSRLNG GEEIFYTTGSRLNGD EEIFYTTGSRLNGDT EIFYTTGSRLNGDTG IFYTTGSRLNGDTGR | IFYTTGSRL | HLA-DRB1*07:01 |
|  | FFYAVDAAY | HLA-C*03:03  HLA-B*35:01  HLA-C*14:02  HLA-A*29:02  HLA-C*12:03  HLA-B*15:02 | 32.02% | LAGEEIFYTTGSRLN GEEIFYTTGSRLNGD EEIFYTTGSRLNGDT |  | HLA-DRB1*01:01 |
|  | FALKGSIEV | HLA-C*03:03  HLA-C*12:03  HLA-A*02:06  HLA-C*15:02  HLA-A*02:01  HLA-C*14:02 | 55.25% | EEIFYTTGSRLNGDT LAGEEIFYTTGSRLN AGEEIFYTTGSRLNG GEEIFYTTGSRLNGD |  | HLA-DPA1*02:01  HLA-DPB1*01:01 |
|  | DASLGGLYY | HLA-C*12:03  HLA-C*03:03  HLA-C*05:01  HLA-B*35:01  HLA-B*15:02  HLA-A*29:02 | 35.75% | RVDYKYSPKLNFSAF ARVDYKYSPKLNFSA VDYKYSPKLNFSAFY VARVDYKYSPKLNFS | YKYSPKLNF | HLA-DRB1*11:01 |
|  | SPKLNFSAF | HLA-C*12:03  HLA-B*07:02  HLA-B*15:02  HLA-B*08:01  HLA-C*03:03  HLA-B*35:01 | 44.27% | RVDYKYSPKLNFSAF VARVDYKYSPKLNFS ARVDYKYSPKLNFSA VDYKYSPKLNFSAFY |  | HLA-DRB1*07:01 |
|  | HANGNLFAL | HLA-C*03:03  HLA-B*15:02  HLA-C*12:03  HLA-B*35:01  HLA-B*39:01  HLA-C*05:01 | 35.09% | KLNFSAFYSYVNLDQ LNFSAFYSYVNLDQG PKLNFSAFYSYVNLD SPKLNFSAFYSYVNL | FSAFYSYVN | HLA-DRB1*04:05 |
|  | IFYTTGSRL | HLA-C*03:03  HLA-C*14:02  HLA-B*15:02  HLA-C*12:03 | 22.69% | KLNFSAFYSYVNLDQ  LNFSAFYSYVNLDQG  SPKLNFSAFYSYVNL  PKLNFSAFYSYVNLD |  | HLA-DRB1*04:04 |
|  | YKYSPKLNF | HLA-C*03:03  HLA-B*15:02  HLA-C*14:02  HLA-C*12:03 | 22.69% | SPKLNFSAFYSYVNL KLNFSAFYSYVNLDQ PKLNFSAFYSYVNLD LNFSAFYSYVNLDQG |  | HLA-DRB1*15:01 |
|  |  |  |  | KLNFSAFYSYVNLDQ |  | HLA-DRB1*04:01 |

Table 4: Most probable predicted epitopes of PEB1A protein interacting with different MHC class I and class II alleles from IEDB analysis tool

| Proteins used | Predicted peptides using IEDB tool | Interacting MHC-I alleles | Population Coverage | Predicted 15 mer peptides using IEDB tool | Core peptide | Interacting MHC-II alleles |
| --- | --- | --- | --- | --- | --- | --- |
| PEB1A | YYQDAIGLL | HLA-C*14:02  HLA-B*15:02  HLA-C*03:03  HLA-C*12:03  HLA-C*07:02  HLA-C*07:01 | 55.69% |  |  |  |
|  | YQDAIGLLV | HLA-A*02:06  HLA-C*12:03  HLA-C*05:01  HLA-A*02:01  HLA-C*08:02 | 53.42% | FSEPYYQDAIGLLVL PYYQDAIGLLVLKEK YQDAIGLLVLKEKKY YYQDAIGLLVLKEKK | YQDAIGLLV | HLA-DRB1*01:01 |
|  |  |  |  | FSEPYYQDAIGLLVL SEPYYQDAIGLLVLK EPYYQDAIGLLVLKE PYYQDAIGLLVLKEK |  | HLA -DRB1*07:01 |
|  | FSVDKSILL | HLA-C*03:03  HLA-C*15:02  HLA-C*07:01  HLA-A*02:06  HLA-B*15:02  HLA-C*12:03 | 41.70% | VDAFSVDKSILLGYV RVDAFSVDKSILLGY DAFSVDKSILLGYVD KRVDAFSVDKSILLG AFSVDKSILLGYVDD FSVDKSILLGYVDDK | FSVDKSILL | HLA-DRB1*07:01 |
|  | MVFRKSLLK | HLA-A*11:01  HLA-A*03:01  HLA-C*12:03  HLA-A*30:01  HLA-A*68:01  HLA-C*07:01 | 56.39% | VDAFSVDKSILLGYV RVDAFSVDKSILLGY KRVDAFSVDKSILLG DAFSVDKSILLGYVD |  | HLA-DRB3*01:01 |
|  | NAKTRGPLL | HLA-C*12:03  HLA-C*03:03  HLA-B*15:02  HLA-C*07:01  HLA-C*06:02 | 49.12% | KRVDAFSVDKSILLG RVDAFSVDKSILLGY |  | HLA-DRB1*01:01 |
|  |  |  |  | VDAFSVDKSILLGYV KRVDAFSVDKSILLG RVDAFSVDKSILLGY DAFSVDKSILLGYVD |  | HLA- DRB1*04:01 |
|  | FSNANAAEG | HLA-C*15:02  HLA-C*03:03  HLA-C*05:01  HLA-C*12:03  HLA-C*07:01 | 45.13% | ACVAFSNANAAEGKL CVAFSNANAAEGKLE VAFSNANAAEGKLES |  | HLA-DQA1*05:01  HLA-DQB1*03:01 |
|  | ATFTITPER | HLA-A*68:01  HLA-C*12:03  HLA-A*31:01  HLA-C*03:03  HLA-A*11:01 | 39.02% | KGFEVDVAKLLAKSI IKGFEVDVAKLLAKS GEIKGFEVDVAKLLA EIKGFEVDVAKLLAK | FEVDVAKLL | HLA- DRB1*01:01 |
|  | QSYGIVTKK | HLA-C*03:03  HLA-C*12:03  HLA-A*11:01  HLA-A*68:01  HLA-A*03:01 | 47.31% | GEIKGFEVDVAKLLA  EIKGFEVDVAKLLAK  IKGFEVDVAKLLAKS  KGFEVDVAKLLAKSI  GFEVDVAKLLAKSIL  FEVDVAKLLAKSILG |  | HLA-DRB3*01:01 |
|  | FEVDVAKLL | HLA-C*03:03  HLA-B*15:02  HLA-B*40:01  HLA-C*12:03 | 26.34% | KGFEVDVAKLLAKSI |  | HLA-DRB1*07:01 |
|  |  |  |  | EIKGFEVDVAKLLAK  GEIKGFEVDVAKLLA  IKGFEVDVAKLLAKS  KGFEVDVAKLLAKSI |  | HLA-DRB1*03:01 |

Table S5: Most probable predicted epitopes of PEB3 protein interacting with different MHC class I and class II alleles from IEDB analysis tool

| Proteins used | Predicted peptides using IEDB tool | Interacting MHC-I alleles | Population Coverage | Predicted 15 mer peptides using IEDB tool | Core peptide | Interacting MHC-II alleles |
| --- | --- | --- | --- | --- | --- | --- |
| PEB3 | MANADVNLY | HLA-B*35:01  HLA-C*05:01  HLA-C*12:03  HLA-C*07:01  HLA-C*03:03  HLA-B*58:01  HLA-C*06:02  HLA-B*53:01  HLA-A*29:02 | 61.56% | IKTIQNFRNNIVAFV KTIQNFRNNIVAFVP | IQNFRNNIV | HLA -DRB1*04:04 |
|  |  |  |  | IKTIQNFRNNIVAFV DIKTIQNFRNNIVAF QDIKTIQNFRNNIVA |  | HLA -DRB1*13:02 |
|  |  |  |  | QDIKTIQNFRNNIVA IKTIQNFRNNIVAFV DIKTIQNFRNNIVAF KTIQNFRNNIVAFVP |  | HLA -DRB1*15:01 |
|  |  |  |  | IKTIQNFRNNIVAFV |  | HLA -DRB1*01:01 |
|  |  |  |  | IKTIQNFRNNIVAFV KTIQNFRNNIVAFVP DIKTIQNFRNNIVAF QDIKTIQNFRNNIVA |  | HLA -DRB1*07:01 |
|  |  |  |  | LVVYRTFNVIAKEGA |  | HLA -DRB1*11:01 |
|  | YRTFNVIAK | HLA-C*03:03  HLA-C*14:02  HLA-C*12:03  HLA-C*07:01  HLA-C*07:02 | 54.57% | KTIQNFRNNIVAFVP TIQNFRNNIVAFVPN IQNFRNNIVAFVPNS QNFRNNIVAFVPNSG | FRNNIVAFV | HLA -DRB1*13:02 |
|  |  |  |  | QNFRNNIVAFVPNSG |  | HLA-DRB1*04:04 |
|  | LTQKGNPLK | HLA-C*12:03  HLA-A*30:01  HLA-C*14:02  HLA-C*03:03  HLA-A*11:01 | 35.88% | KTIQNFRNNIVAFVP IQNFRNNIVAFVPNS TIQNFRNNIVAFVPN QNFRNNIVAFVPNSG |  | HLA- DRB1*01:01 |
|  |  |  |  | KTIQNFRNNIVAFVP  TIQNFRNNIVAFVPN  IQNFRNNIVAFVPNS  QNFRNNIVAFVPNSG |  | HLA- DRB3*01:01 |
|  | FRNNIVAFV | HLA-C*06:02  HLA-C*12:03  HLA-C*07:01  HLA-C*05:01 | 47.63% |  |  |  |

Table S6: Most probable predicted epitopes of CadF protein interacting with different MHC class I and class II alleles from IEDB analysis tool

| **Protein used** | **Predicted peptides using IEDB tool** | **Interacting MHC-I alleles** | **Population Coverage** | **Predicted 15 mer peptides using IEDB tool** | **Core peptide** | **Interacting MHC-II alleles** |
| --- | --- | --- | --- | --- | --- | --- |
| CadF | FRLSDSLAL | HLA-B*39:01  HLA-C*03:03  HLA-B*15:02  HLA-C*14:02  HLA-C*07:02 | 34.76% | AGVKFRLSDSLALRL  VKFRLSDSLALRLET  GVKFRLSDSLALRLE  YGAGVKFRLSDSLAL  GAGVKFRLSDSLALR  KFRLSDSLALRLETR  FRLSDSLALRLETRD | FRLSDSLAL | HLA-DRB1*07:01  HLA-DRB1*01:01  HLA-DRB5*01:01  HLA-DRB1*09:01  HLA-DRB1*04:01 |
|  | NYFEGNLDM | HLA-C*14:02  HLA-C*07:01  HLA-C*06:02  HLA-C*12:03  HLA-C*07:02  HLA-B*15:02 | 60.72% | PTLNYNYFEGNLDMD | NYFEGNLDM | HLA-DRB1*04:05 |
|  |  |  |  | TLNYNYFEGNLDMDN |  | HLA-DRB1*04:05 |
|  |  |  |  | LNYNYFEGNLDMDNR |  | HLA-DRB1*04:05 |
|  |  |  |  | TPTLNYNYFEGNLDM |  | HLA-DRB1*04:05 |
|  |  |  |  | YNYFEGNLDMDNRYA |  | HLA-DRB1*04:05 |
|  |  |  |  | NYNYFEGNLDMDNRY |  | HLA-DRB1*04:05 |
|  | NRYAPGIRL | HLA-C*07:01  HLA-C*06:02  HLA-C*03:03  HLA-B*15:02  HLA-C*14:02  HLA-C*07:02  HLA-C*12:03 | 65.69% |  |  |  |
|  | FYGLAGGGY | HLA-C*14:02  HLA-C*12:03  HLA-B*15:02  HLA-C*03:03  HLA-A*29:02 | 25.70% | EKFYFYGLAGGGYED  KFYFYGLAGGGYEDF  GEKFYFYGLAGGGYE  VGEKFYFYGLAGGGY  FYFYGLAGGGYEDFS  YFYGLAGGGYEDFSN | FYGLAGGGY | HLA-DQA1*05:01  HLA-DQB1*03:01  HLA-DRB1*01:01 |
|  | YFYGLAGGG | HLA-C*03:03  HLA-C*12:03  HLA-C*14:02 | 20.74% | EKFYFYGLAGGGYED | YFYGLAGGG | HLA-DQA1*05:01  HLA-DQB1*03:01 |
|  |  |  |  | KFYFYGLAGGGYEDF |  | HLA-DQA1*05:01  HLA-DQB1*03:01 |
|  |  |  |  | GEKFYFYGLAGGGYE |  | HLA-DQA1*05:01  HLA-DQB1*03:01 |
|  |  |  |  | VGEKFYFYGLAGGGY |  | HLA-DQA1*05:01  HLA-DQB1*03:01  HLA-DRB1*01:01 |
|  |  |  |  | FYFYGLAGGGYEDFS |  | HLA-DQA1*05:01  HLA-DQB1*03:01 |
|  |  |  |  | YFYGLAGGGYEDFSN |  | HLA-DQA1*05:01  HLA-DQB1*03:01 |
|  | FGHYGAGVK | HLA-C*14:02  HLA-C*12:03  HLA-C*03:03 | 20.74% | KSGGFGHYGAGVKFR  NKSGGFGHYGAGVKF | FGHYGAGVK | HLA-DQA1*05:01  HLA-DQB1*03:01 |
|  |  |  |  | SGGFGHYGAGVKFRL  GGFGHYGAGVKFRLS  KSGGFGHYGAGVKFR  NKSGGFGHYGAGVKF |  | HLA-DRB5*01:01  HLA-DRB5*01:01  HLA-DRB5*01:01  HLA-DRB5*01:01 |
|  |  |  |  | GSRAYNQKLSERRAK  SRAYNQKLSERRAKS  RAYNQKLSERRAKSV | YNQKLSERR | HLA-DRB5*01:01 |
|  |  |  |  | IGSRAYNQKLSERRA  AYNQKLSERRAKSVA  YNQKLSERRAKSVAN |  | HLA-DRB5*01:01 |
|  |  |  |  | ITRTYLSAIKGIDVG  TRTYLSAIKGIDVGE  RTYLSAIKGIDVGEK  TYLSAIKGIDVGEKF | LSAIKGIDV | HLA-DRB1*07:01  HLA-DRB1*01:01 |
|  |  |  |  | PTLNYNYFEGNLDMD  TLNYNYFEGNLDMDN  LNYNYFEGNLDMDNR  TPTLNYNYFEGNLDM  NYNYFEGNLDMDNRY  YNYFEGNLDMDNRYA | YNYFEGNLD | HLA-DRB1*04:05 |
|  |  |  |  | ELEKYGVEKSRIKTV  LEKYGVEKSRIKTVG  NELEKYGVEKSRIKT  EKYGVEKSRIKTVGY  KYGVEKSRIKTVGYG  YGVEKSRIKTVGYGQ | YGVEKSRIK | HLA-DRB5*01:01 |
|  | SSNDTKEGR | HLA-C*12:03  HLA-C*07:01  HLA-C*05:01  HLA-C*15:02  HLA-A*31:01 | 42.09% |  |  |  |
|  | PVEPREGAL | HLA-C*03:03  HLA-C*05:01  HLA-C*12:03  HLA-C*07:02  HLA-B*15:02  HLA-C*14:02 | 47.10% |  |  |  |
|  | FEITPTLNY | HLA-C*12:03  HLA-B*15:02  HLA-C*03:03  HLA-B*44:03  HLA-A*29:02 | 27.99% |  |  |  |
|  | ERYDTILEG | HLA-C*07:01,  HLA-C*12:03,  HLA-C*14:02,  HLA-C*03:03,  HLA-C*06:02 | 50.02% |  |  |  |

Table S7: Most probable predicted epitopes of Cia protein interacting with different MHC class I and class II alleles from IEDB analysis tool

| **Protein used** | **Predicted peptides using IEDB tool** | **Interacting MHC-I alleles** | **PopulationCoverage** | **Predicted 15 mer peptides using IEDB tool** | **Core peptide** | **Interacting MHC-II alleles** |
| --- | --- | --- | --- | --- | --- | --- |
| Cia | FYYDLYEKI | HLA-C*14:02 HLA-C*03:03 HLA-C*12:03 HLA-A*23:01 | 25.05% | VKFYYDLYEKIGNEI | FYYDLYEKI | HLA-DPA1*03:01 HLA-DPB1*04:02 |
|  |  |  |  | KFYYDLYEKIGNEID FYYDLYEKIGNEIDN |  | HLA-DPA1*01:03 HLA-DPB1*02:01 |
|  |  |  |  | KEFVKFYYDLYEKIG |  | HLA-DPA1*02:01 HLA-DPB1*01:01 HLA-DPA1*03:01 HLA-DPB1*04:02 HLA-DPA1*01:03 HLA-DPB1*02:01 HLA-DPA1*01 HLA-DPB1*04:01 |
|  |  |  |  | EFVKFYYDLYEKIGN |  | HLA-DPA1*01:03 HLA-DPB1*02:01 HLA-DPA1*03:01 HLA-DPB1*04:02 HLA-DPA1*01 HLA-DPB1*04:01 HLA-DPA1*02:01 HLA-DPB1*01:01 |
|  |  |  |  | FVKFYYDLYEKIGNE |  | HLA-DPA1*01:03 HLA-DPB1*02:01 HLA-DPA1*03:01 HLA-DPB1*04:02 HLA-DPA1*01 HLA-DPB1*04:01 HLA-DPA1*02:01 HLA-DPB1*01:01 |
|  |  |  |  | VKFYYDLYEKIGNEI |  | HLA-DPA1*01:03 HLA-DPB1*02:01  HLA-DPA1*01 HLA-DPB1*04:01  HLA-DPA1*02:01 HLA-DPB1*01:01 |
|  | FFYHEQDDL | HLA-C*03:03,  HLA-C*14:02,  HLA-C*12:03,  HLA-C*07:02,  HLA-B*15:02 | 41.19% | GLINFFYHEQDDLIM GGLINFFYHEQDDLI LINFFYHEQDDLIMP INFFYHEQDDLIMPV | FYYDLYEKI | HLA-DPA1*02:01 HLA-DPB1*01:01 |
|  | YYTEGLIHL | HLA-C*14:02 HLA-B*15:02 HLA-C*07:02 HLA-C*03:03 HLA-C*12:03 | 41.19% | VRPYYTEGLIHLSLL  RPYYTEGLIHLSLLF  EVRPYYTEGLIHLSL  DEVRPYYTEGLIHLS | YYTEGLIHL | HLA-DPA1*03:01 HLA-DPB1*04:02 HLA-DPA1*02:01 HLA-DPB1*01:01 |
|  | LIMPVFHEL | HLA-A*02:06 HLA-A*02:01 HLA-C*15:02 HLA-C*12:03 HLA-A*68:02 | 50.86% | DDLIMPVFHELIKRA QDDLIMPVFHELIKR EQDDLIMPVFHELIK HEQDDLIMPVFHELI | LIMPVFHEL | HLA-DPA1*02:01 HLA-DPB1*01:01 HLA-DPA1*01:03 HLA-DPB1*02:01 |
|  | FYQEKIWKR | HLA-C*14:02 HLA-C*12:03 HLA-A*31:01 HLA-C*07:02 | 36.69% | RNILFYQEKIWKRVY NILFYQEKIWKRVYE GRNILFYQEKIWKRV ILFYQEKIWKRVYEV | FYQEKIWKR | HLA-DRB5*01:01 |
|  | LIFENNNLK | HLA-C*12:03 HLA-A*11:01 HLA-C*07:01 HLA-A*68:01 | 43.56% | SEVLIFENNNLKINF FESEVLIFENNNLKI ESEVLIFENNNLKIN EVLIFENNNLKINFD | LIFENNNLK | HLA-DRB5*01:01 |
|  | FMKISSEVF | HLA-B*15:01 HLA-B*15:02 HLA-C*12:03 HLA-C*14:02 | 22.56% | AKTKPFMKISSEVFD KTKPFMKISSEVFDK TKPFMKISSEVFDKE KPFMKISSEVFDKEF | FMKISSEVF | HLA-DRB1*07:01 HLA-DRB1*01:01 HLA-DRB5*01:01 |
|  |  |  |  | FMKISSEVFDKEFLD PFMKISSEVFDKEFL |  | HLA-DRB1*07:01 HLA-DRB1*01:01 |
|  | IFAFINFVY | HLA-A*29:02 HLA-C*03:03 HLA-C*12:03 HLA-B*35:01 | 27.81% | KKIFAFINFVYENAK | IFAFINFVY | HLA-DPA1*02:01 HLA-DPB1*05:01  HLA-DPA1*02:01  HLA-DPB1*01:01 |
|  |  |  |  | KAGKKIFAFINFVYE |  | HLA-DPA1*02:01  HLA-DPB1*05:01  HLA-DPA1*01  HLA-DPB1*04:01  HLA-DPA1*02:01  HLA-DPB1*01:01  HLA-DPA1*01:03  HLA-DPB1*02:01 |
|  |  |  |  | AGKKIFAFINFVYEN |  | HLA-DPA1*02:01  HLA-DPB1*05:01  HLA-DPA1*02:01  HLA-DPB *01:01 |
|  |  |  |  | GKKIFAFINFVYENA |  | HLA-DPA1*02:01  HLA-DPB *05:01  HLA-DPA1*02:01 |
|  |  |  |  | KIFAFINFVYENAKT |  | HLA-DPA1*02:01  HLA-DPB1*05:01 |
|  |  |  |  | IFAFINFVYENAKTK |  | HLA-DRB5*01:01 |
|  | KAYIEYFEK | HLA-C*12:03 HLA-C*03:03 HLA-A*11:01 HLA-A*30:01 HLA-A*31:01 | 37.60% | SSEEEKAYIEYFEKL | KAYIEYFEK | HLA-DPA1*01 HLA-DPB1*04:01 HLA-DPA1*01:03 HLA-DPB1*02:01 |
|  | YTHAVALEW | HLA-B*58:01 HLA-C*14:02 HLA-C*15:02 HLA-C*12:03 HLA-B*57:01 HLA-C*03:03 | 30.13% |  |  |  |
|  | TMNQSGFFK | HLA-A*11:01 HLA-A*03:01 HLA-A*68:01 HLA-C*12:03 HLA-C*05:01 HLA-A*31:01 | 50.67% |  |  |  |
|  |  |  |  | EEKAYIEYFEKLKLA | AYIEYFEKL | HLA-DPA1*01:03 HLA-DPB1*02:01 HLA-DPA1*03:01 HLA-DPB1*04:02 HLA -DPA1*01 HLA DPB1*04:01 |
|  |  |  |  | EEEKAYIEYFEKLK |  | HLA -DPA1*01:03 HLA-DPB1*02:01 HLA -DPA1*02:01 HLA DPB1*01:01 HLA- DPA1*03:01 HLA-DPB1*04:02 HLA- DPA1*01 HLA-DPB1*04:01 |
|  |  |  |  | SEEEKAYIEYFEKLK |  | HLA- DPA1*01:03 HLA-DPB1*02:01 HLA- DPA1*01 HLA-DPB1*04:01 |
|  |  |  |  | EKAYIEYFEKLKLAF |  | HLA- DPA1*01:03 HLA-DPB1*02:01 HLA- DPA1*01:03 HLA-DPB1*02:01 HLA- DPA1*01:03 HLA-DPB1*02:01 |
|  |  |  |  | KAYIEYFEKLKLAFC |  | HLA- DPA1*01:03 HLA-DPB1*02:01 |
|  |  |  |  | AYIEYFEKLKLAFCE |  | HLA- DPA1*01:03  HLA-DPB1*02:01 |
|  |  |  |  | DVGEYFRSLISLSEL | FRSLISLSE | HLA-DRB1*04:05 HLA-DRB1*01:01 HLA-DRB1*04:04 HLA-DRB1*04:01 HLA-DRB1*11:01 HLA-DRB5*01:01 |
|  |  |  |  | VGEYFRSLISLSELK |  | HLA-DRB5*01:01 HLA-DRB1*11:01 HLA-DRB1*04:01 HLA-DRB1*04:04 HLA-DRB1*01:01 HLA-DRB1*04:05 |
|  |  |  |  | EYFRSLISLSELKQD |  | HLA-DRB1*04:05 HLA-DRB1*01:01 HLA-DRB1*04:04 HLA-DRB1*04:01 HLA-DRB1*11:01 HLA-DRB5*01:01 |
|  |  |  |  |  |  |  |
|  |  |  |  | FYGAELKGLFSAQVV | LKGLFSAQV | HLA-DRB1*01:01 HLA-DRB1*09:01 HLA-DRB1*04:04 HLA-DRB1*04:05 HLA-DRB1*07:01 |
|  |  |  |  | GAELKGLFSAQVVPN |  | HLA-DRB1*07:01 HLA-DRB1*04:05 HLA-DRB1*09:01 HLA-DRB1*09:01 HLA-DRB1*01:01 |
|  |  |  |  | YGAELKGLFSAQVVP |  | HLA-DRB1*01:01 HLA-DRB1*09:01 HLA-DRB1*04:04 HLA-DRB1*04:05 HLA-DRB1*07:01 |
|  |  |  |  | AELKGLFSAQVVPND |  | HLA-DRB1*07:01 HLA-DRB1*04:05 HLA-DRB1*09:01 HLA-DRB1*04:04 HLA-DRB1*01:01 |


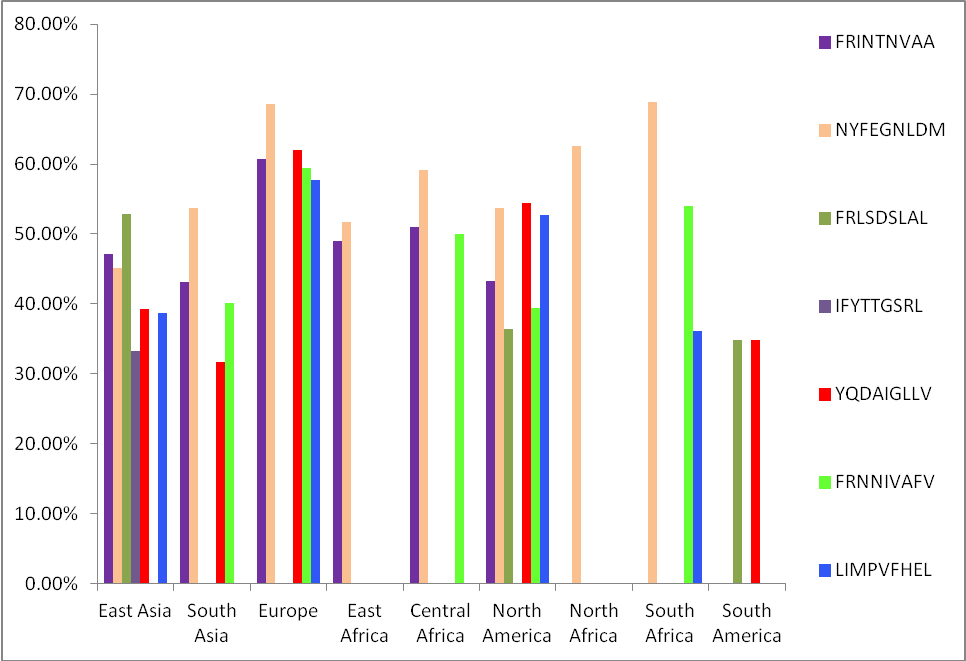


Figure S1: Population coverage by MHC Class I restricted epitopes predicted from FlaA, CAdF, MOMP, PEB1A, PEB3, Cia proteins of *Campylobacter jejuni*.


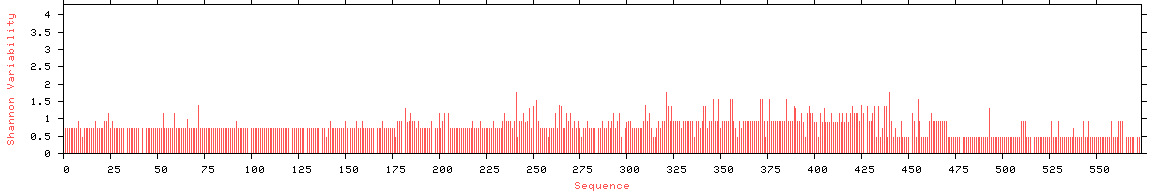


a)

b)


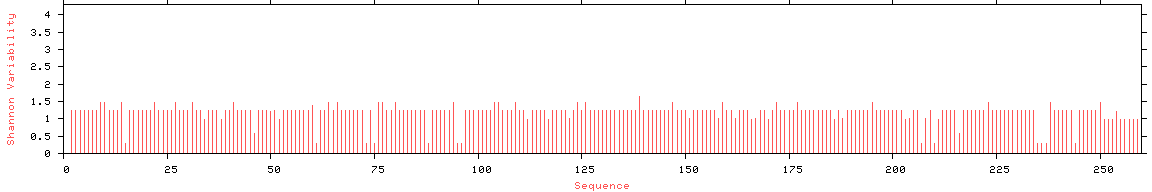


c)


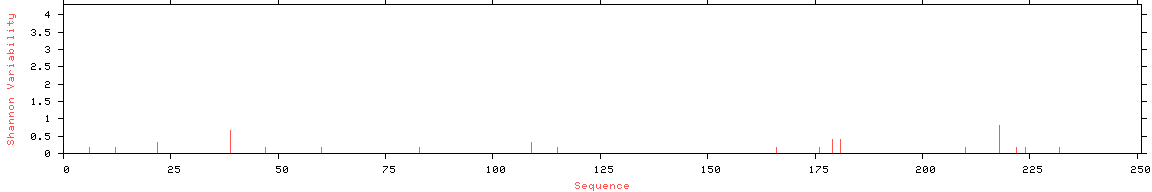


d)

**
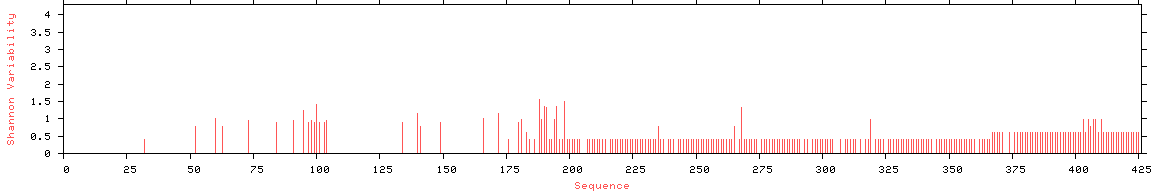
**

e)

**
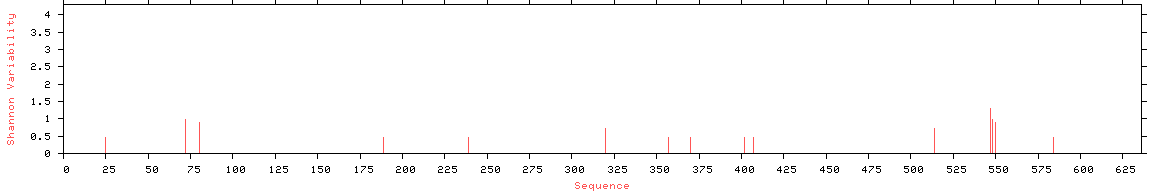
**

f)

*
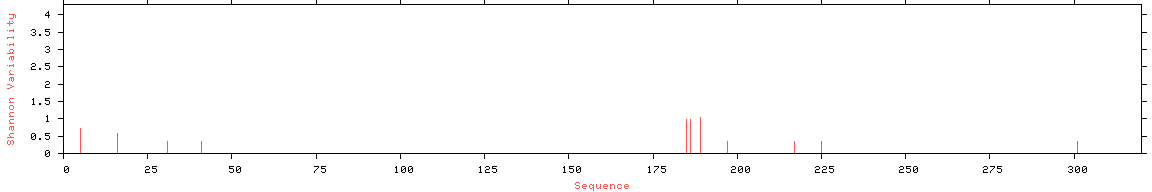
*

Figure S2: The protein variability index of the epitopes from a) FlaA, b) PEB1A, c) PEB3, d) MOMP, e) Cia and f) CadF protein was determined by using PVS server. The epitope positions are indicated by arrow signs. The conservancy threshold was 1.0 in this analysis. X axis indicates the amino acid position in sequences and Y axis indicates the Shannon variability.

84-25 MKKIFLCLGLASVLFGADNNVKFEITPTLNYNYFEGNLDMDNRYAPGIRLGYHFDDFWLD

11168-BN148 MKKIFLCLGLASVLFGADNNVKFEITPTLNYNYFEGNLDMDNRYAPGIRLGYHFDDFWLD

CG8421 MKKIFLCLGLASVLFGADNNVKFEITPTLNYNYFEGNLDMDNRYAPGIRLGYHFDDFWLD

CF93-6 MKKIFLCLGLASVLFGADNNVKFEITPTLNYNYFEGNLDMDNRYAPGIRLGYHFDDFWLD

IA3902 MKKIFLCLGLASVLFGADNNVKFEITPTLNYNYFEGNLDMDNRYAPGIRLGYHFDDFWLD

subsp. MKKIFLCLGLASVLFGADNNVKFEITPTLNYNYFEGNLDMDNRYAPGIRLGYHFDDFWLD

260.94 MKKIFLCLGLASVLFGADNNVKFEITPTLNYNYFEGNLDMDNRYAPGIRLGYHFDDFWLD

700819 MKKIFLCLGLASVLFGADNNVKFEITPTLNYNYFEGNLDMDNRYAPGIRLGYHFDDFWLD

RM1221 MKKIFLCLGLASVLFGADNNVKFEITPTLNHNYFEGNLDMDNRYAPGIRLGYHFDDFWLD

CJM1cam MKKIFLCLGLASVLFGADNNVKFEITPTLNYNYFEGNLDMDNRYAPGIRLGYHFDDFWLD

M1 MKKIFLCLGLASVLFGADNNVKFEITPTLNYNYFEGNLDMDNRYAPGIRLGYHFDDFWLD

81116 MKKIFLCLGLASVLFGADNNVKFEITPTLNYNYFEGNLDMDNRYAPGIRLGYHFDDFWLD

81-176 MKKILLCLGLASVLFSADNNVKFEITPTLNYNYFEGNLDMDNRYAPGIRLGYHFDDFWLD

BAA-1458 MKKILLCLGLASVLFGADNNVKFEITPTLNYNYFEGNLDMGNRYAPGIRLGYHFDDFWLD

****:**********.**************:*********.*******************

84-25 QLEFGLEHYSDVKYTNTNKTTDITRTYLSAIKGIDVGEKFYFYGLAGGGYEDFSNAAYDN

11168-BN148 QLEFGLEHYSDVKYTNTNKTTDITRTYLSAIKGIDVGEKFYFYGLAGGGYEDFSNAAYDN

CG8421 QLEFGLEHYSDVKYTNTNKTTDITRTYLSAIKGIDVGEKFYFYGLAGGGYEDFSNAAYDN

CF93-6 QLEFGLEHYSDVKYTNTNKTTDITRTYLSAIKGIDVGEKFYFYGLAGGGYEDFSNAAYDN

IA3902 QLEFGLEHYSDVKYTNTNKTTDITRTYLSAIKGIDVGEKFYFYGLAGGGYEDFSNAAYDN

subsp. QLEFGLEHYSDVKYTNTNKTTDITRTYLSAIKGIDVGEKFYFYGLAGGGYEDFSNAAYDN

260.94 QLEFGLEHYSDVKYTNTNKTTDITRTYLSAIKGIDVGEKFYFYGLAGGGYEDFSNAAYDN

700819 QLEFGLEHYSDVKYTNTNKTTDITRTYLSAIKGIDVGEKFYFYGLAGGGYEDFSNAAYDN

RM1221 QLEFGLEHYSDVKYTNTNKTTDITRTYLSAIKGIDVGEKFYFYGLAGGGYEDFSNAAYDN

CJM1cam QLEFGLEHYSDVKYTNTNKTTDITRTYLSAIKGIDVGEKFYFYGLAGGGYEDFSNAAYDN

M1 QLEFGLEHYSDVKYTNTNKTTDITRTYLSAIKGIDVGEKFYFYGLAGGGYEDFSNAAYDN

81116 QLEFGLEHYSDVKYTNTNKTTDITRTYLSAIKGIDVGEKFYFYGLAGGGYEDFSNAAYDN

81-176 QLEFGLEHYSDVKYTNTNKTTDITRTYLSAIKGIDVGEKFYFYGLAGGGYEDFSNAAYDN

BAA-1458 QLEFGLEHYSDVKYTNTNKTTDITRTYLSAIKGIDVGEKFYFYGLAGGGYEDFSNAAYDN

************************************************************

84-25 KSGGFGHYGAGVKFRLSDSLALRLETRDQINFNHANHNWVSTLGISFGFGGKKEKAVEEV

11168-BN148 KSGGFGHYGAGVKFRLSDSLALRLETRDQINFNHANHNWVSTLGISFGFGGKKEKAVEEV

CG8421 KSGGFGHYGAGVKFRLSDSLALRLETRDQINFNHANHNWVSTLGISFGFGGKKEKAVEEV

CF93-6 KSGGFGHYGAGVKFRLSDSLALRLETRDQINFNHANHNWVSTLGISFGFGGKKEKAVEEV

IA3902 KSGGFGHYGAGVKFRLSDSLALRLETRDQINFNHANHNWVSTLGISFGFGGKKEKAVEEV

subsp. KSGGFGHYGAGVKFRLSDSLALRLETRDQINFNHANHNWVSTLGISFGFGGKKEKAVEEV

260.94 KSGGFGHYGAGVKFRLSDSLALRLETRDQINFNHANHNWVSTLGISFGFGGKKEKAVEEV

700819 KSGGFGHYGAGVKFRLSDSLALRLETRDQINFNHANHNWVSTLGISFGFGGKKEKAVEEV

RM1221 KSGGFGHYGAGVKFRLSDSLALRLETRDQINFNHANHNWVSTLGISFGFGGKKEKAVEEV

CJM1cam KSGGFGHYGAGVKFRLSDSLALRLETRDQINFNHANHNWVSTLGISFGFGGKKEKAVEEV

M1 KSGGFGHYGAGVKFRLSDSLALRLETRDQINFNHANHNWVSTLGISFGFGGKKEKAVEEV

81116 KSGGFGHYGAGVKFRLSDSLALRLETRDQINFNHANHNWVSTLGISFGFGGKKEKAVEEV

81-176 KSGGFGHYGAGVKFRLSDSLALRLETRDQINFNHANHNWVSTLGISFGFGGKKEKAVEEV

BAA-1458 KSGGFGHYGAGVKFRLSDSLALRLETRDQINFNHANHNWVSTLGISFGFGGKKEKAVEEV

************************************************************

84-25 ADTRATPQAKCPVEPREGALLDENGCEKTISLEGHFGFDKTTINPTFQEKIKEIAKVLDE

11168-BN148 ADTRATPQAKCPVEPREGALLDENGCEKTISLEGHFGFDKTTINPTFQEKIKEIAKVLDE

CG8421 ADTRATPQAKCPVEPREGALLDENGCEKTISLEGHFGFDKTTINPTFQEKIKEIAKVLDE

CF93-6 ADTRATPQAKCPVEPREGALLDENGCEKTISLEGHFGFDKTTINPTFQEKIKEIAKVLDE

IA3902 ADTRATPQAKCPVEPREGALLDENGCEKTISLEGHFGFDKTTINPTFQEKIKEIAKVLDE

subsp. ADTRATPQAKCPVEPREGALLDENGCEKTISLEGHFGFDKTTINPTFQEKIKEIAKVLDE

260.94 ADTRATPQAKCPVEPREGALLDENGCEKTISLEGHFGFDKTTINPTFQEKIKEIAKVLDE

700819 ADTRATPQAKCPVEPREGALLDENGCEKTISLEGHFGFDKTTINPTFQEKIKEIAKVLDE

RM1221 ADTRATPQVKCPVEPREGALLDENGCEKTISLEGHFGFDKTTINPTFQEKIKEIAKVLDE

CJM1cam ADTRPAPQTKCPVEPREGALLDENGCEKTISLEGHFGFDKTTINPTFQEKIKEIAKVLDE

M1 ADTRPAPQTKCPVEPREGALLDENGCEKTISLEGHFGFDKTTINPTFQEKIKEIAKVLDE

81116 ADTRPAPQTKCPVEPREGALLDENGCEKTISLEGHFGFDKTTINPTFQEKIKEIAKVLDE

81-176 ADTRPAPQAKCPVEPREGALLDENGCEKTISLEGHFGFDKTTINPTFQEKIKEIAKVLDE

BAA-1458 ADTRPAPQAKCPVEPRGGALLDENGCEKTISLEGHFDFDKTTINLTFQEKIKEIAKVLDE

****.:**.******* *******************.******* ***************

84-25 NERYDTILEGHTDNIGSRAYNQKLSERRAKSVANELEKYGVEKSRIKTVGYGQDNPRSSN

11168-BN148 NERYDTILEGHTDNIGSRAYNQKLSERRAKSVANELEKYGVEKSRIKTVGYGQDNPRSSN

CG8421 NERYDTILEGHTDNIGSRAYNQKLSERRAKSVANELEKYGVEKSRIKTVGYGQDNPRSSN

CF93-6 NERYDTILEGHTDNIGSRAYNQKLSERRAKSVANELEKYGVEKSRIKTVGYGQDNPRSSN

IA3902 NERYDTILEGHTDNIGSRAYNQKLSERRAKSVANELEKYGVEKSRIKTVGYGQDNPRSSN

subsp. NERYDTILEGHTDNIGSRAYNQKLSERRAKSVANELEKYGVEKSRIKTVGYGQDNPRSSN

260.94 NERYDTILEGHTDNIGSRAYNQKLSERRAKSVANELEKYGVEKSRIKTVGYGQDNPRSSN

700819 NERYDTILEGHTDNIGSRAYNQKLSERRAKSVANELEKYGVEKSRIKTVGYGQDNPRSSN

RM1221 NERYDTILEGHTDNIGSRAYNQKLSERRAKSVANELEKYGVEKSRIKTVGYGQDNPRSSN

CJM1cam NERYDTILEGHTDNIGSRAYNQKLSERRAKSVANELEKYGVEKSRIKTVGYGQDNPRSSN

M1 NERYDTILEGHTDNIGSRAYNQKLSERRAKSVANELEKYGVEKSRIKTVGYGQDNPRSSN

81116 NERYDTILEGHTDNIGSRAYNQKLSERRAKSVANELEKYGVEKSRIKTVGYGQDNPRSSN

81-176 NERYDTILEGHTDNIGSRAYNQKLSERRAKSVANELEKYGVEKSRIKTVGYGQDNPRSSN

BAA-1458 NERYDTILEGHTDNIGSRAYNQKLSERRAKSVANELEKYGVEKSRIKTVGYGQDNPRSSN

************************************************************

84-25 DTKEGRADNRRVDAKFILR

11168-BN148 DTKEGRADNRRVDAKFILR

CG8421 DTKEGRADNRRVDAKFILR

CF93-6 DTKEGRADNRRVDAKFILR

IA3902 DTKEGRADNRRVDAKFILR

subsp. DTKEGRADNRRVDAKFILR

260.94 DTKEGRADNRRVDAKFILR

700819 DTKEGRADNRRVDAKFILR

RM1221 DTKEGRADNRRVDAKFILR

CJM1cam DTKEGRADNRRVDAKFILR

M1 DTKEGRADNRRVDAKFILR

81116 DTKEGRADNRRVDAKFILR

81-176 DTKEGRADNRRVDAKFILR

BAA-1458 NTKEGRADNRRVDAKFILR

:******************

Figure S3: Multiple Sequence Alignment of CadF protein done by CLUSTAL. The conserved epitope sequence is shown in yellow.
